# Surgical Outcomes After Risk-Reducing Mastectomy Among *BRCA1* and *BRCA2* Carriers

**DOI:** 10.1001/jamanetworkopen.2026.2574

**Published:** 2026-04-03

**Authors:** Rebecca Wiberg, Signe Hägglund, Barbro Numan Hellquist, Anna Rosén, Annika Idahl, Maria Mani, Svetlana Bajalica-Lagercrantz, Hans Ehrencrona, Per Karlsson, Niklas Loman, Malin Sund

**Affiliations:** 1 Department of Diagnostics and Intervention, Plastic Surgery and Surgery, Umeå University, Umeå, Sweden; 2 Department of Diagnostics and Intervention, Oncology, Umeå University, Umeå, Sweden; 3 Department of Clinical Sciences, Obstetrics and Gynecology, Umeå University, Umeå, Sweden; 4 Department of Surgical Sciences, Plastic and Reconstructive Surgery, Uppsala University, Uppsala, Sweden; 5 Department of Oncology-Pathology, Karolinska Institutet, Stockholm, Sweden; 6 Department of Clinical Genetics and Genomics, Hereditary Cancer Unit, Karolinska University Hospital, Stockholm, Sweden; 7 Department of Laboratory Medicine, Division of Clinical Genetics, Lund University, Lund, Sweden; 8 Department of Clinical Genetics, Pathology and Molecular Diagnostics, Skåne University Hospital, Lund, Sweden; 9 Department of Oncology, Institute of Clinical Sciences, Sahlgrenska Academy, University of Gothenburg, Gothenburg, Sweden; 10 Department of Hematology, Oncology and Radiation Physics, Skåne University Hospital, Malmö, Sweden; 11 Department of Clinical Sciences, Lund University, Lund, Sweden; 12 Department of Surgery, University of Helsinki and Helsinki University Hospital, Helsinki, Finland

## Abstract

**Importance:**

Long-term follow-up studies regarding the safety of risk-reducing mastectomy (RRM) in terms of cancer risk and surgical complications among women with germline pathogenic variants (gPVs) in *BRCA1* or *BRCA2* (*BRCA1/2*) are scarce.

**Objective:**

To analyze cancer risk and surgical complications after RRM.

**Design, Setting, and Participants:**

This nationwide cohort study investigated 1208 Swedish women with a confirmed gPV in *BRCA1/2* without previous breast cancer identified through the Swedish Cancer Genetic Units between March 31, 1994, and January 2, 2019. Data were extracted from the National Patient Care Register, the Cancer Register, and the Cause of Death Register. Women were followed up from the date of RRM (RRM group) or genetic testing (no RRM group) until breast cancer diagnosis, death, emigration, or end of follow-up (December 31, 2023).

**Main Outcomes and Measures:**

Breast cancer incidence was calculated per 10 000 person-years, with women undergoing RRM contributing person-years to the no RRM group until RRM, and women with occult breast cancer contributing breast cancer cases to the no RRM group.

**Results:**

In the RRM group (507 women; median age at RRM, 39.7 years [range, 19.6-72.1 years]), 1 woman developed breast cancer, corresponding to a breast cancer incidence of 2 cases per 10 000 person-years. In the no RRM group (701 women; median age at genetic testing, 50.6 years [range, 4.3-93.6 years]), 112 women developed breast cancer, corresponding to a breast cancer incidence of 162 cases per 10 000 person-years. At RRM, 17 of 507 women (3.4%) received a diagnosis of occult breast cancer. In the RRM group, 296 of 507 women (58.4%) underwent simple mastectomy, 143 of 507 (28.2%) underwent nipple-sparing mastectomy, and 68 of 507 (13.4%) underwent skin-sparing mastectomy. Most women (382 of 507 [75.3%]) underwent implant-based breast reconstruction, with only 73 of 507 (14.4%) undergoing autologous tissue reconstruction with or without implants. Early major surgical postoperative complications associated with reoperation occurred in 19 of 507 women (3.7%).

**Conclusions and Relevance:**

In this cohort study of 1208 women with a gPV in *BRCA1/2*, the risk of developing breast cancer or early major surgical complications after RRM was very low. The low occurrence of primary breast cancer precluded meaningful statistical comparisons between different RRM techniques.

## Key Points

**Question:**

Is risk-reducing mastectomy a safe procedure for *BRCA1* or *BRCA2* carriers in terms of cancer risk and surgical complications?

**Findings:**

In this cohort study of 1208 women, breast cancer was diagnosed in 1 of 507 women undergoing risk-reducing mastectomy (incidence, 2 cases per 10 000 person-years) and 112 of 701 women not undergoing risk-reducing mastectomy (incidence, 162 cases per 10 000 person-years). Nineteen of 507 women (3.7%) experienced a major early postoperative complication.

**Meaning:**

This study suggests that risk-reducing mastectomy for *BRCA1* and *BRCA2* carriers is safe.

## Teaser

This cohort study analyzes cancer risk and surgical complications after risk-reducing mastectomy for *BRCA1* and *BRCA2* carriers.

## Introduction

Breast cancer accounts for 1 in 4 cancer cases among women.^1^ Germline pathogenic variants (gPVs) in the breast cancer predisposition genes *BRCA1* (OMIM: 113705) and *BRCA2* (OMIM: 600185) (henceforth, *BRCA1/2*) are associated with approximately 2.4% of all breast cancers.^2,3^ Women with gPVs in *BRCA1* have a mean cumulative lifetime risk of breast cancer of 72% and a mean cumulative lifetime risk of ovarian cancer of 44%; women with gPVs in *BRCA2* have a mean cumulative lifetime risk of breast cancer of 69% and a mean cumulative lifetime risk of ovarian cancer of 17%.^4^ National and international guidelines therefore recommend intensified surveillance with annual breast imaging and risk-reducing salpingo-oophorectomy (RRSO) for women with identified gPVs in *BRCA1/2*.^5,6^ In addition, these women are informed about the possibility of undergoing risk-reducing mastectomy (RRM). Bilateral RRM decreases the incidence of breast cancer by 90% or more,^7,8^ with primary breast cancer observed in approximately 1.9% of women after bilateral RRM after 3 to 14 years of follow-up.^9,10,11,12,13^

There is an ongoing debate about how much subcutaneous tissue should be resected to perform an adequate RRM while leaving viable skin flaps with an acceptable aesthetic outcome. To allow immediate breast reconstruction (IBR) after RRM, skin-sparing mastectomy (SSM) or nipple-sparing mastectomy (NSM) is often performed. The presumed increased oncologic risk of SSM and NSM in comparison with simple mastectomy is attributed to potential residual breast tissue in the skin flaps and/or the nipple-areolar complex. Studies have, however, shown comparable oncologic risks after SSM or NSM and simple mastectomy,^14,15^ although few studies report long-term oncologic safety. The main objective of this study was to analyze surgical outcomes after RRM, including analysis of breast cancer incidence, mastectomy and breast reconstruction techniques, and postoperative complications.

## Methods

This cohort study was performed according to the Declaration of Helsinki^16^ and ethical guidelines of the Swedish Research Council. Informed consent was waived due to the register-based design. In accordance with the ethical guidelines of the Swedish Research Council and the General Data Protection Regulation, the requirement for informed consent may be waived for this type of research, provided that the study has received ethical approval and that the data are processed in a pseudonymized manner, ensuring that individual participants cannot be identified. Ethical approval was obtained from the Regional Ethical Board in Lund. The study is reported in accordance with the Strengthening the Reporting of Observational Studies in Epidemiology (STROBE) reporting guideline.

**Figure 1.**
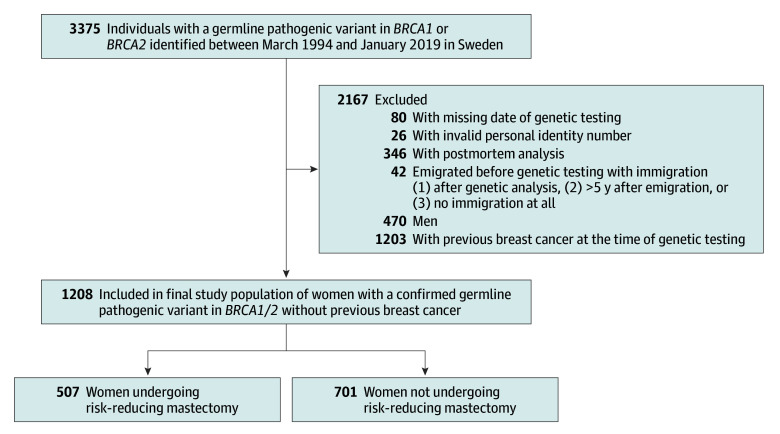
Flowchart for Study Inclusion

This is a nationwide register-based observational cohort study of Swedish women with confirmed gPVs in *BRCA1/2*. In 1994, genetic screening for suspected hereditary breast cancer and ovarian cancer started in Sweden. Women were identified through the Swedish Cancer Genetic Units, and the testing was gradually centralized to 1 laboratory (BRCA-lab, Lund University, Lund, Sweden). This laboratory also performed predictive testing, although also done at some other clinical genetics departments. The present cohort was identified through the BRCA-lab and also includes individuals identified at the Department of Clinical Genetics in Gothenburg. The cohort consists of 3375 individuals with confirmed gPVs in *BRCA1/2* between March 31, 1994, and January 2, 2019. Individuals were excluded for the following reasons: missing date of genetic testing; postmortem analysis; emigration before genetic testing with immigration (1) after genetic analysis, (2) more than 5 years after emigration, or (3) no immigration at all; age younger than 18 years at the end of follow-up; male sex; and previous breast cancer (Figure 1). Two groups were generated: women undergoing RRM (RRM group) and those not undergoing RRM (no RRM group) during the follow-up period. Follow-up was defined as the time from RRM (RRM group) or genetic testing (no RRM group) until a breast cancer diagnosis, death, emigration, or end of follow-up, whichever event came first. Women undergoing RRM contributed person-years to the no RRM group until RRM, and women with occult breast cancer contributed breast cancer cases to the no RRM group. Follow-up was until December 31, 2022 (breast cancer), and December 31, 2023 (surgeries and survival).

Data linkages were made for the gPV-positive individuals with the following Swedish registers: the National Patient Register,^17^ the National Cancer Register,^18^ and the National Cause of Death Register.^19^ Diagnosis codes from the *International Classification of Disease* were used: *International Statistical Classification of Diseases and Related Health Problems, Tenth Revision* (*ICD-10*; from 1997 onward), *International Classification of Diseases, Ninth Revision* (*ICD-9*; 1987-1996), *International Classification of Diseases, Eighth Revision* (*ICD-8*; 1969-1986), *International Classification of Diseases, Seventh Revision* (*ICD-7*) (1964-1968), *International Classification of Diseases for Oncology*, *Third Edition* (*ICD-O-3*; from 2004 onward for the northern region and from 2005 onward for the rest of Sweden), and *International Classification of Diseases for Oncology*, Second *Edition* (*ICD-O-2*) (from 1993 to 2003 or 2004). Surgical procedure codes were based on the National Classification of Health Care Interventions (national abbreviation: KVÅ, from 1997 onward) and the National Classification of Operations (national abbreviation: KOP, 1963-1996) (eTable in Supplement 1).

RRM was defined as a mastectomy without a previous breast cancer diagnosis and without a sentinel node biopsy or other axillary procedure performed at the time of the mastectomy. Women who received a diagnosis of breast cancer at or within 90 days of RRM were defined as having occult breast cancer. Women who received a diagnosis of breast cancer more than 90 days after RRM were defined as having primary breast cancer after RRM. Breast cancer was defined as invasive breast cancer or ductal carcinoma in situ (DCIS). However, prior to the introduction of *ICD-O-2* (1993), the morphology code for breast cancer in situ did not distinguish between DCIS and lobular carcinoma in situ (LCIS), nor did the topographic breast cancer code 170 (*ICD-7*). To be consistent, the topographic breast cancer code 170 (*ICD-7*) was used to exclude women with previous breast cancer, resulting in the incorrect exclusion of some women with LCIS.

A major surgical postoperative complication (msPOC) was defined as the registering of at least 1 prespecified surgical procedure code for bleeding, wound, infectious, and/or unspecified complication in association with the index inpatient episode, or a new inpatient episode (readmission) within 30 days of RRM.

### Statistical Analysis

Descriptive statistics of clinical variables were presented as numbers with percentages, mean (SD) values, and median values with ranges (minimum to maximum), stratified by *BRCA1/2* status. When considering the time between genetic testing and RRM, only women undergoing RRM after genetic testing were included. Breast cancer incidence was measured through breast cancer diagnoses per 10 000 person-years of observation. The Wilcoxon rank sum test and the Fisher exact test were used to test for significant differences between groups because normal distribution could not always be assumed and because some groups were small. The Charlson Comorbidity Index (CCI), calculated with the adaptation by Armitage and van der Meulen^20^ and Brusselaers and Lagergren,^21^ was used for evaluation of comorbidities and stratified by having 1 or more comorbidity diagnosis or having none, with data extracted from the National Patient Register. The CCI was presented as percentages, with odds ratios (ORs), 95% CIs, and *P* values. Statistical significance was considered reached with a 2-sided *P* < .05. Statistical analyses were performed using R statistical software, version 4.4.0 (R Project for Statistical Computing).

## Results

### Study Population

The inclusion criteria were met by 1208 women (median age at genetic testing, 43.7 years [range, 4.3-93.6 years]) (Figure 1; Table 1). There were 507 women who underwent RRM and 701 who did not undergo RRM during the median follow-up period of 9.4 person-years (range, 0.02-29.8 person-years) per individual, corresponding to a total follow-up time of 11 957 person-years for the entire cohort. The median age at genetic testing was 13.5 years younger in the RRM group than in the no RRM group (37.1 years [range, 16.8-70.6 years] vs 50.6 years [4.3-93.6 years]; *P* < .001). The median age at RRM was 39.7 years (range, 19.6-72.1 years), with most women (341 of 507 [67.3%]) undergoing surgery between 31 and 50 years of age. The median time from genetic testing to RRM was 1.8 years (range, 0.04-21.7 years), with *BRCA2* gPV carriers undergoing RRM sooner after genetic testing in comparison with *BRCA1* gPV carriers (median, 1.5 years [range, 0.1-21.7 years] vs 2.0 years [range, 0.04-17.3 years]; *P* = .01). Women aged 40 years or older at RRM were more likely to undergo RRM sooner after genetic testing compared with women younger than 40 years (median time between genetic testing and RRM, 1.3 years [range, 0.1-9.8 years] vs 2.6 years [range, 0.04-21.7 years]; *P* < .001). A lower proportion of women undergoing RRM than women in the no RRM group had a comorbidity diagnosis at genetic testing (16.6% [84 of 507] vs 40.5% [284 of 701] had ≥1 comorbidity diagnosis; OR, 3.43 [95% CI, 2.58-4.59]; *P* < .001). In the RRM group, 228 of 507 women (45.0%) also underwent RRSO; 134 of 228 (58.8%) underwent RRSO before RRM and 94 of 228 (41.2%) underwent RRSO after RRM. In the no RRM group, 240 of 701 women (34.2%) underwent RRSO. Characteristics of the study cohort are presented in Table 1.

**Table 1.** Characteristics of the Study Cohort

Characteristic	No. (%)			*P* value^b^
	Total	*BRCA1* ^a^	*BRCA2* ^a^	
Women without a previous breast cancer at the time of genetic testing	1208 (100)	904 (100)	302 (100)	
Women not undergoing RRM (no RRM group)	701 (58.0)	515 (57.0)	184 (60.9)	.25
Women undergoing RRM	507 (42.0)	389 (43.0)	118 (39.1)	
Age at genetic testing, y				
Mean (SD)	44.8 (15.7)	44.1 (15.4)	46.9 (16.6)	.02
Median (range)	43.6 (4.3-93.6)	43.1 (4.3-90.7)	44.8 (16.8-93.6)	
Age at RRM, y				
Mean (SD)	41.2 (10.6)	40.7 (10.4)	42.6 (11.4)	.09
Median (range)	39.7 (19.6-72.1)	38.6 (20.7-72.1)	42.4 (19.6-70.2)	
≤30	64 (12.6)	52 (13.7)	12 (10.2)	
31-40	191 (37.7)	153 (39.3)	38 (32.2)	
41-50	150 (29.6)	107 (27.5)	43 (36.4)	
>50	100 (19.7)	75 (19.3)	25 (21.2)	
Time from genetic testing to RRM, y^c^				
Mean (SD)	3.1 (3.2)	3.3 (3.3)	2.5 (2.8)	.01
Median (range)	1.8 (0.04-21.7)	2.0 (0.04-17.3)	1.5 (0.1-21.7)	
Time from RRM to end of follow-up, y				
Mean (SD)	9.8 (6.6)	9.8 (6.5)	9.8 (6.9)	.88
Median (range)	8.6 (0.0-38.0)	8.8 (0.0-37.1)	8.3 (0.0-38.0)	
Time from genetic testing to end of follow-up in the no RRM group, y^d^				
Mean (SD)	5.8 (5.3)	5.8 (5.3)	5.9 (5.2)	.72
Median (range)	4.4 (0.02-27.6)	4.2 (0.04-26.7)	5.1 (0.02-27.6)	
Total follow-up time, person-years per woman				
Mean (SD)	10.5 (6.3)	10.7 (6.4)	10.2 (5.7)	.33
Median (range)	9.4 (0.02-29.8)	9.6 (0.1-29.8)	8.9 (0.02-27.6)	

Abbreviations: gPV, germline pathogenic variant; RRM, risk-reducing mastectomy.

^a^ Including women carrying a gPV in either *BRCA1* or *BRCA2* (2 women with both *BRCA1* and *BRCA2* excluded).

^b^ *BRCA1* vs *BRCA2*.

^c^ Estimated only for women undergoing risk-reducing mastectomy after genetic testing (11 excluded).

^d^ Including women not undergoing risk-reducing mastectomy during the follow-up, and women undergoing risk-reducing mastectomy from the time of genetic testing until risk-reducing mastectomy.

### Breast Cancer Incidence

In the RRM group, 1 woman developed primary breast cancer after a median follow-up of 8.6 years (range, 0.0-38.0 years). The total amount of at-risk time in the RRM group was 5037 person-years, corresponding to a breast cancer incidence of 2 cases per 10 000 person-years (Table 2). Stratified by RRM technique, the total amount of person-years was 3108 for simple mastectomy, 615 for SSM, and 1244 for NSM. In the no RRM group, 112 women developed primary breast cancer after a median follow-up of 4.4 years (range, 0.02-27.6 years). The total amount of at-risk time in the no RRM group was 6920 person-years, corresponding to a breast cancer incidence of 162 cases per 10 000 person-years. Of the 112 women who developed breast cancer in the no RRM group, 92 had invasive cancers and 20 had DCIS. During follow-up, 7 women received a diagnosis of LCIS.

**Table 2.** Breast Cancer Incidence^a^

Group	No. of women			Person-years of follow-up			No. of breast cancer cases			Breast cancer incidence per 10 000 person-years		
	*BRCA1*	*BRCA2*	Total^b^	*BRCA1*	*BRCA2*	Total^b^	*BRCA1*	*BRCA2*	Total	*BRCA1*	*BRCA2*	Total
RRM	389	118	507	3865	1172	5037	1	0	1	3	NA	2
No RRM	515	184	701	5177	1720	6920	87	25	112	168	145	162
Total	904	302	1208	9042	2892	11957	88	25	113	97	86	95

Abbreviations: NA, not applicable; RRM, risk-reducing mastectomy.

^a^ Breast cancer incidence during the follow-up period, including occult breast cancers identified at the time of RRM in the no RRM group.

^b^ The total may differ from *BRCA1* or *BRCA2* separately because 2 women with both *BRCA1* and *BRCA2* were included in the total.

Occult breast cancer was identified in 17 of 507 women (3.4%) and 20 breasts at the time of RRM, of which 5 of 20 (25.0%) were invasive breast cancer, 14 of 20 (70.0%) were DCIS, and 1 of 20 (5.0%) was Paget disease. Furthermore, occult LCIS was identified in 9 women and 11 breasts at the time of RRM. The median age at RRM was 9.9 years older among women with occult cancer than women without occult cancer (49.0 years [range, 31.7-63.9 years] vs 39.1 years [range, 19.6-72.1 years]; *P* = .003). Occult breast cancer was detected in 7 of 255 (2.7%) of the first 50% of women undergoing RRM and 10 of 252 (4.0%) of the remaining 50% (split point between 2014 and 2015), with no difference between the time periods (OR, 0.68 [95% CI, 0.22-2.03]; *P* = .47). The precise time at which preoperative imaging was implemented in clinical routine is not known; thus, to illustrate how an altered preoperative imaging routine might have changed the outcome of occult breast cancer over time, 2015 was selected as the dividing point, as 50% of the women had undergone RRM before 2015 and the remaining 50% after 2015. After mastectomy, no women with occult findings developed a new breast cancer or died during follow-up. Women with occult breast cancer received adequate adjuvant treatment based on the tumor characteristics.

### Mastectomy and Reconstruction Techniques

**Figure 2.**
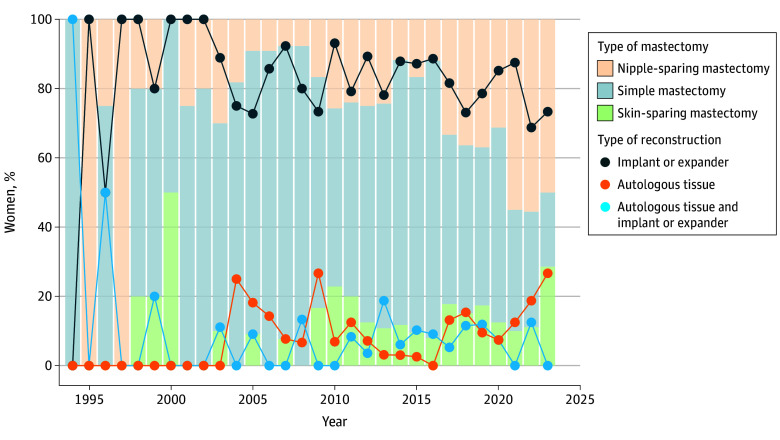
Mastectomy and Breast Reconstruction Techniques Over Time Type of mastectomy in women undergoing risk-reducing mastectomy, including simple mastectomy, skin-sparing mastectomy, and nipple-sparing mastectomy, and type of immediate breast reconstruction, including implant-based reconstruction, autologous reconstruction, and implant in combination with autologous tissue. Surgical techniques are presented as percentages of the total surgeries performed from 1994 to 2023, excluding 2 mastectomies and 1 breast reconstruction that were performed prior to 1994.

Of 507 women undergoing RRM, more than half (296 of 507 [58.4%]) underwent simple mastectomy, 143 (28.2%) underwent NSM, and 68 (13.4%) underwent SSM. The proportions of NSM and SSM seemed to increase over time (Figure 2). The median age at RRM was 40.1 years (range, 19.6-72.1 years) for women undergoing simple mastectomy, 38.2 years (range, 21.4-68.7 years) for women undergoing SSM, and 40.1 years (range, 20.1-71.6 years) for women undergoing NSM. Most women (456 of 507 [89.9%]) underwent an IBR, most of the breasts were reconstructed with implants (382 of 456 [83.8%]). Only 43 of 456 women (9.4%) underwent reconstruction with autologous tissue, 30 of 456 women (6.6%) underwent reconstruction with implant combined with autologous tissue, and 1 of 456 women (0.2%) underwent another type of reconstruction. The median age at IBR was 3.9 years younger among women undergoing implant-based compared with autologous tissue reconstruction (38.4 years [range, 18.9-72.1 years] vs 42.3 years [range, 24.2-62.3 years]; *P* = .02). Use of autologous tissue reconstruction seemed to increase over time (Figure 2). Of 51 women undergoing RRM without IBR, 16 (31.4%) later underwent breast reconstruction. The median age for women not undergoing IBR was 40.5 years (range, 18.9-72.1 years). The trends for surgical techniques in RRM and IBR over time are presented in Figure 2. The CCI at RRM did not differ among women undergoing RRM with or without IBR (25.4% [116 of 456] vs 29.4% [15 of 51] had ≥1 comorbidity diagnosis; OR, 0.82 [95% CI, 0.42-1.67]; *P* = .61).

### Major Surgical Postoperative Complications

Any msPOC associated with reoperation during a 30-day interval after RRM occurred in 19 of 507 women (3.7%). Among identified msPOCs, bleeding complications occurred in 15 of 507 women (3.0%), wound complications in 1 of 507 women (0.2%), infectious complications in 2 of 507 women (0.4%), and unspecified msPOCs in 2 of 507 women (0.4%). One woman experienced both a bleeding complication and an unspecified complication. Of all women experiencing early msPOCs after RRM, 3 had implant removal due to an msPOC. No women died within 30 days of RRM. Women with or without IBR had similar rates of msPOCs (17 of 456 [3.7%] vs 2 of 51 [3.9%]). The CCI at RRM did not differ between women experiencing any msPOC within 30 days after RRM and those who did not (21.1% [4 of 19] vs 26.0% [127 of 488] had ≥1 comorbidity diagnosis; OR, 0.76 [95% CI, 0.18-2.44]; *P* = .79). Any msPOC occurred in 11 of 382 women with implant-based reconstruction (2.9%), 3 of 43 women with autologous reconstruction (7.0%), 3 of 30 women with combined implant and autologous tissue reconstruction (10.0%), and 2 of 51 women without IBR (3.9%).

## Discussion

RRM was associated with reduced risk of breast cancer by more than 90%,^7,8,12,13,22,23^ which was also confirmed by our study. With a median follow-up of 8.6 years, only 1 woman developed primary breast cancer in the RRM group, corresponding to a breast cancer incidence of 2 cases per 10 000 person-years. In a meta-analysis from 2016 including 2555 participants, the occurrence of post-RRM breast cancer in cases compared with controls corresponded to a relative risk of 0.11 (95% CI, 0.03-0.32), confirming a significant reduction in breast cancer risk.^8^ In a prospective study including 1654 *BRCA1/2* gPV carriers without breast cancer, Metcalfe et al^24^ showed that 5 of 827 women (0.6%) undergoing RRM received a diagnosis of breast cancer during a mean follow-up of 6.3 years, in comparison with 100 of 827 women (12.1%) who did not undergo RRM. In 2019, a national multicenter cohort in the Netherlands^25^ evaluated breast cancer incidence among *BRCA1/2* gPV carriers without cancer with breasts and ovaries in situ. In line with our results, they reported breast cancer in 8 of 1128 *BRCA1/2* gPV carriers (0.7%) after bilateral RRM compared with 412 of 1729 *BRCA1/2* gPV carriers (23.8%) staying under surveillance during a median follow-up of 10 years.^25^

Achieving complete removal of all breast tissue when performing a mastectomy is an unattainable goal due to the risk of flap necrosis if skin circulation is compromised. The unknown oncologic risk with residual breast tissue has made the use of NSM and, to some degree, SSM among women with gPVs in *BRCA1/2* controversial. In the prospective SKINI (Radicality of Skin-Sparing and Nipple-Sparing Mastectomy) trial,^26^ intraoperative biopsies were taken from the skin envelope of 160 women undergoing therapeutic mastectomy or RRM, with residual breast tissue detected in 68.9% of women who underwent NSM vs 40.4% of women who underwent SSM (*P* < .001). Nevertheless, clinical studies comparing SSM or NSM with simple mastectomy have shown comparable subsequent risks of breast cancer.^8,14,15,27,28,29,30^ Jakub et al^31^ reviewed the outcome of 346 *BRCA1/2* gPV carriers undergoing risk-reducing NSM and found no breast cancers after surgery. They concluded that NSM should be offered as a breast cancer risk-reducing strategy to women with gPVs in *BRCA1/2*; however, the follow-up in their study was merely 34 months. In this study, the low occurrence of primary breast cancer precluded meaningful statistical comparisons between different RRM techniques.

Occult breast cancer is occasionally identified in women younger than 40 years,^9,24,32^ with the prevalence of occult malignant or premalignant lesions in RRM specimens varying from 1.6% to 10% in the literature.^9,24,30,32^ In our study, occult breast cancer was identified in 17 women and 20 breasts, corresponding to an overall prevalence of 3.4%. Notable, only 5 of the occult tumors were invasive breast cancer. Based on a review from 2024,^33^ occult invasive malignant neoplasms should be detected in less than 1% of RRMs performed on *BRCA1/2* gPV carriers with normal preoperative imaging. The proportion of occult breast cancer, however, remained constant in different time periods in this cohort, despite changes in national guidelines, which currently recommend preoperative imaging within 3 months before RRM.^6^ This finding could not be further investigated within the present study, as the setup lacked access to medical records and, accordingly, preoperative imaging findings.

In our cohort, 58.4% of all women in the RRM group underwent simple mastectomy, 28.2% underwent NSM, and 13.4% underwent SSM. As previously shown,^28,34,35,36^ implant-based IBR was by far the most common reconstruction technique after RRM. Although autologous reconstruction is becoming increasingly popular,^37,38^ this finding was not fully mirrored in the current cohort, with only a small proportion of women undergoing such reconstruction. After RRM, up to 50% of all women experience more than 1 early (<30 days) complication, with the most common being partial skin necrosis or epidermolysis requiring conservative treatment.^9,28^ NSM is increasingly performed with the intent of obtaining superior cosmetic outcomes in comparison with SSM and simple mastectomy. However, the risk of skin flap necrosis is higher with NSM,^39,40^ thus increasing the risk of infections and implant loss. The high degree of complications and the need for further corrective surgical procedures should be emphasized during the preoperative consultation because up to 20% of women undergoing RRM are dissatisfied with the surgery, with adverse symptoms, complications, and insufficient information or support being frequently reported causes of dissatisfaction.^41^ In the present study, 19 of 507 women (3.7%) had an msPOC requiring reoperation within 30 days from RRM. Due to the register-based study design, the association between various surgical techniques and msPOCs requiring only conservative treatment could not be studied. Furthermore, the low number of msPOCs did not allow for comparisons regarding msPOCs and surgical techniques.

### Strengths and Limitations

This study has several strengths. It comprises a nationwide cohort of women with confirmed gPVs in *BRCA1/2*, excluding other high-risk groups, based on register data with high reliability, with a long-term follow-up, and minimal loss to follow-up, and is of high clinical value.

This study also has some limitations. Although the coverage of *BRCA1/2* gPV carriers was high, it does not completely correspond to all women without breast cancer with identified gPVs in *BRCA1/2* in Sweden, as some predictive testing is performed at local laboratories. Also, because the topographic Cancer Register code 170 (*ICD-7*) does not distinguish between invasive and some preinvasive lesions, women with LCIS might have been incorrectly classified when creating the study population. In addition, the no RRM group retained heterogeneity in follow-up time, including women not undergoing RRM, as well as women undergoing RRM until the date of surgery. Relatively low numbers of SSMs and NSMs were reported, which might not fully mirror reality because the type of RRM was based on the surgical procedure code rather than a review of medical records. Furthermore, the msPOCs reported in this study were based on surgical procedure codes and not diagnosis codes, limiting the msPOCs to those requiring surgery. Subsequently, other important medical complications, such as deep venous thrombosis and pulmonary embolism, were not identified. Extramammary tumors with possible origin from the breast might not have been reported in the Cancer Register as breast cancer; however, the Cancer Register is a validated registry with accurate coding.^42^

## Conclusions

This nationwide cohort study shows that RRM is a safe procedure with very low rates of breast cancer and complications. The low occurrence of primary breast cancer precluded meaningful statistical comparisons between different RRM techniques.

## Notes

Supplement 1. **eTable. **Included Diagnosis and Surgical Procedure Codes

Supplement 2. **Nonauthor Collaborators.** Members of the Swedish BRCA Study Group

Supplement 3.
Data Sharing Statement
