# Supplementary material for: Surgical Outcomes After Risk-Reducing Mastectomy Among BRCA1 and BRCA2 Carriers
Source: JAMA Netw Open. 2026 Apr 3;9(4):e262574. doi: 10.1001/jamanetworkopen.2026.2574 (PMC13049491; doi:10.1001/jamanetworkopen.2026.2574)
Supplement: Supplement 1. — eTable. Included Diagnosis and Surgical Procedure Codes [file jamanetwopen-e262574-s001.pdf]

Supplementary Online Content

Wiberg R, Hägglund S, Numan Hellquist B, et al; Swedish BRCA Study Group. Surgical outcomes after risk-reducing mastectomy among *BRCA1* and *BRCA2* carriers. *JAMA Netw Open.* 2026;9(3):e262574. doi:10.1001/jamanetworkopen.2026.2574

eTable. Included Diagnosis and Surgical Procedure Codes

This supplementary material has been provided by the authors to give readers additional information about their work.

© 2026 Wiberg R et al. *JAMA Network Open.*

eTable. Included Diagnosis and Surgical Procedure Codes. Included codes obtained from the Swedish Patient Register and the Swedish Cancer Register.
Abbreviations: ICD=International Classification of Disease; DCIS=ductal carcinoma in situ; LCIS=lobular carcinoma in situ; KVÅ=Classification of Health Care Interventions (national abbreviation); KOP=Classification of Operations (national abbreviation); NA=not available.

eTable. Included Diagnosis and Surgical Procedure Codes

	Diagnosis and surgical procedure code	
**Cancer diagnosis**	**ICD-9**	**ICD-7**
Breast cancer	174	170
Ovarian cancer	1588, 1589, 183, 1952, 1953	158, 175, 1993, 1994
**Morphology of breast cancer**	**ICD-O/3.2**	**ICD-O/2**
DCIS	85002	85002
Morbus Paget	85403	85403
LCIS	85202	85202
**Surgical procedure**	**KVÅ**	**KOP**
**Risk-reducing mastectomy[a]**		
Simple mastectomy	HAC20, HAC99	3820
Skin-sparing mastectomy	HAC15	3812
Nipple-sparing mastectomy	HAC10	3813
**Breast reconstruction**		
Implant-based	HAD00, HAD10, HAE00	3853, 3854
With autologous tissue	HAE10	3852
With implant and autologous tissue	HAE05	3855
Other reconstruction	HAE99	3858
**Bilateral salpingo-oophorectomy[b]**		
Bilateral procedures (one code required)	LAE20, LAE21, LAF10, LAF11, LAF30	7011, 7021, 7031
Unilateral procedures (two codes required)	LAE10, LAE11, LAF00, LAF01, LAF20	7010, 7020, 7030, 7032
**Axillary surgery**		
Exploration of lymph nodes	VXA20, PJA10, PJA20	NA
Radical evacuation of axillary lymph nodes	VXK26, PJD52	8871

© 2026 Wiberg R et al. *JAMA Network Open.*

Extirpation of axillary lymph nodes	VXK21, PJD42	8870, 8880
**Surgical procedure**	**KVÅ**	**KOP**
**Complications**		
Hemorrhage complication	HWD00, HWE00, HWF00	3896
Wound complication	HWA00	3895
Infection	HWB00, HWC00	3897
Unspecified complication	HWW99	3898
Implant explantation	HAD50	3860

**Charlson Comorbidity Index**	**Weight**	**ICD-10**	**ICD-9**	**ICD-8**
Myocardial infarction	1	I252	412	412
Acute myocardial infarction	1	I21–I23	410	410
Congestive heart failure	1	I11, I13, I255, I42, I43, I50, I517	402, 425, 428, 429D	427, 428
Peripheral vascular disease	1	I70–I73, I770, I771, K558, K559, Z958, Z959, K551, R02	440–447, V43E	440–445
Cerebrovascular disease	1	G45, G46, I6	430–438, 362D	430–438
Dementia	1	F00–F03, G30, G31, A810, F051	290, 294B	290
Chronic pulmonary disease	1	I26, I27, J40–J47, J60–J67, J684, J701, J703	490–496, 500–505, 416, 506E	490–493, 515–518
Rheumatic disease	1	M05, M06, M32–M36, M09, M120, M315	710–714, 725	710–712, 734
Liver disease	1	K70, K71, B18, I85, I864, I982, K721, K729, K76, R162, Z944	070, 456A–456C, 571–573	070, 456, 571, 573
Diabetes mellitus	1	E10, E14	250	250
Hemiplegia, paraplegia	1	G81, G83, G114	342–344	344
Renal disease	1	I12, I13, N01, N03, N05, N07, N08, N18, N25, Z49, Z940, Z992	403, 404, 581–583, 585–588, V42A, V45B	403, 404, 582, 583
Acute renal disease	1	N171, N172, N19	584	580, 581
Malignancy	1	C0, C1, C20, C21, C23–C26, C30–C34, C37–C41, C45–C58, C6, C70–C76, C80–C85, C90–C97, C43, C88	14–16, 170–172, 174–179, 18, 190–195, 200–208	14–16, 170–172, 174–179, 18, 190–195, 200–207

© 2026 Wiberg R et al. *JAMA Network Open.*

Metastatic tumors	1	C77–C79	196–199	196–199
AIDS, HIV	1	B20–B24	279K	NA

[a] with the absence of any previous or simultaneous breast cancer diagnosis, sentinel node biopsy or other axillary procedure.
[b] risk-reducing if absence of previous or simultaneous (at or within 3 months of salpingo-oophorectomy) ovarian cancer diagnosis.

© 2026 Wiberg R et al. *JAMA Network Open.*
