# Supplementary material for: Surgical Outcomes After Risk-Reducing Mastectomy Among BRCA1 and BRCA2 Carriers
Source: JAMA Netw Open. 2026 Apr 3;9(4):e262574. doi: 10.1001/jamanetworkopen.2026.2574 (PMC13049491; doi:10.1001/jamanetworkopen.2026.2574)
Supplement: Supplement 2. — Nonauthor Collaborators. Members of the Swedish BRCA Study Group [file jamanetwopen-e262574-s002.pdf]

Supplemental Online Content: Nonauthor Collaborators

*First name, last name, and suffix (if applicable) are required and will appear in PubMed.

*Group Name(s): The Swedish BRCA study group							
***First Name and Middle Initial(s)**	***Last Name**	***Suffix (eg, Jr, III)**	Academic Degrees	Institution	Location (city, state/province, country)	Role or Contribution, eg, chair, principal investigator	Group (if more than 1 Group listed in the byline) and/or Subgroup (eg, Steering Committee)
Åke	Borg		PhD	Division of Oncology, Department of Clinical Sciences Lund, Lund University	Lund, Sweden	Member	
Anna	Öfverholm		MD, PhD	Institute of Clinical Sciences, Department of Oncology, Sahlgrenska Academy, Gothenburg University	Gothenburg, Sweden	Member	
Anna	von Wachenfeldt		MD, PhD	Department of Clinical Science and Education, Karolinska Institute, Södersjukhuset	Stockholm, Sweden	Member	
Christina	Edwinsdotter Ardnor		MD	Department of Radiation Sciences/Oncology, Umeå University	Umeå, Sweden	Member	
Ekaterina	Kuchinskaya		MD, PhD	Department of Clinical Pathology and Clinical Genetics, and Department of Clinical and Experimental Medicine, Linköping University	Linköping, Sweden	Member	
Johanna	Rantala		PhD	Department of Molecular Medicine and Surgery, Karolinska Institutet, Stockholm, Sweden and Department of Clinical Genetics, Karolinska University Hospital	Stockholm, Sweden	Member	
Ylva	Karlsson		MD, PhD	Department of Immunology, Genetics and Pathology, Science for Life Laboratory, Uppsala University	Uppsala, Sweden	Member	
