# Supplementary material for: Surgical Outcomes After Risk-Reducing Mastectomy Among BRCA1 and BRCA2 Carriers
Source: JAMA Netw Open. 2026 Apr 3;9(4):e262574. doi: 10.1001/jamanetworkopen.2026.2574 (PMC13049491; doi:10.1001/jamanetworkopen.2026.2574)
Supplement: Supplement 3. — Data Sharing Statement [file jamanetwopen-e262574-s003.pdf]

Data Sharing Statement

Wiberg. Surgical Outcomes After Risk-Reducing Mastectomy Among BRCA1 and BRCA2 Carriers. *JAMA Netw Open*. Published April 03, 2026.
doi:10.1001/jamanetworkopen.2026.2574

Data
Data available: No

Additional Information
Explanation for why data not available: The data that support the findings of this study are not openly available due to reasons of sensitivity but are available from the corresponding author upon reasonable request.
